# Relationship between Postoperative Anterior Chamber Depth and Refraction Based on the Haptic Fix Position in Intraocular Lens Intrascleral Fixation

**DOI:** 10.3390/jcm12051815

**Published:** 2023-02-24

**Authors:** Santaro Noguchi, Shunsuke Nakakura, Asuka Noguchi, Hitoshi Tabuchi

**Affiliations:** Department of Ophthalmology, Saneikai Tsukazaki Hospital, Himeji 671-1227, Japan

**Keywords:** intrascleral intraocular lens fixation, refractive error, iris capture

## Abstract

The aim of this study was to analyze the refraction and iris capture tendency regarding the fixation position with respect to the intrascleral fixation (ISF) of intraocular lenses. Consecutive patients who underwent ISF 1.5 mm (ISF 1.5, 45 eyes) and 2.0 mm (ISF 2.0, 55 eyes) from the corneal limbus with NX60, as well as those who underwent normal phacoemulsification with in-the-bag ZCB00V (ZCB, 50 eyes), were enrolled. The anterior chamber depth (post-op ACD), the estimated ACD when using the SRK/T (post-op ACD—predicted ACD), and the refractive error (post-op MRSE, and the predicted MRSE) were all calculated. In addition, the postoperative iris capture was also investigated. The post-op MRSE—predicted MRSE values were: −0.59, 0.02, and 0.00 D (ISF 1.5, ISF 2.0, and ZCB) (*p* < 0.05, between ISF 1.5 vs. ISF 2.0 and ZCB); the post-op ACD values were: 4.00, 4.17, and 4.29 mm (*p* < 0.05, ISF 1.5 vs. ZCB); and the post-op ACD—predicted ACD values were: −2.03, −1.98, and −1.60 mm (*p* < 0.05, between ZCB vs. ISF 1.5 and ISF 2.0). The iris capture occurred in four eyes with regard to ISF 1.5 and three eyes with ISF 2.0 (*p* = 0.52). Moreover, ISF 2.0 possessed 0.6D hyperopia and 0.17 mm deeper anterior chamber depth. The refractive error of ISF 2.0 was less than that of ISF 1.5. Lastly, no significant iris capture onset was noted between ISF 1.5 mm and 2.0 mm.

## 1. Introduction

The concept of intrascleral intraocular lens (IOL) fixation was first reported by Gabor and Pavlidis in 2007 [1]. In addition, the non-flapped double-needle technique [2] and flanged intrascleral IOL fixation technique [3] were reported by Yamane et al. Similar to normal cataract operations, the simplification of techniques has resulted in increasingly steady results and fewer complications. This has been achieved in addition to the aim of improving visual acuity [4,5]. However, when performing intrascleral fixation (ISF), the complication of iris capture that is sometimes observed with sutured IOL fixation can also occur in certain cases with sutureless IOL fixation [6]. As iris capture can occur in the long run, the possibility of this complication must be considered during the postoperative monitoring of sutureless IOL fixations.

It must be noted that not all cases of intrascleral fixation are dependent on the lens capsule fixation. Due to the fact that most of the cases are concerned with the dislocation of the lens and with zonal loss, the anterior chamber depth of the distance, as well as the lens thickness, cannot be used as the preoperative calculation formulas. Therefore, it is currently considered most useful to use the SRK/T formula, which can obtain the predicted refraction from the corneal refractive power and the axial length of the eye [7,8,9]. However, the location of the IOL fixation is expected to be different with regard to intrascleral fixation when compared to the in-the-bag fixation. Although it has been reported that hyperopia of around 0.8 D was observed within cases of scleral fixation, when using the SRK/T formula, detailed studies on the scleral fixation position and refraction error have not yet been conducted [7,8]. The position of the haptic fixation to the sclera is generally determined on the basis of the distance from the corneal limbus at the discretion of the surgeon. The expected refractive outcome is currently calculated with an in-the-bag formula, such as SRK/T, and then through the adjustment of the IOL power that is based on this value. Naturally, in those performing ISF, when the site of haptic fixation in the sclera is far from the limbus, the IOL will be fixated at a deeper point, thereby resulting in hyperopia. Alternatively, if the IOL is fixated closer to the limbus, it will be at a shallower site, thus resulting in myopia. However, no detailed information is available regarding how the fixation position alters the postoperative refraction and the resulting refractive error. As a deeper IOL fixation position results in a longer distance between the iris and the IOL, the frequency of iris capture is also expected to be lower. However, no data are available comparing iris capture with the IOL fixation position that were performed using the Yamane technique. Indeed, the IOL fixation position appears to possess very important implications for ISF surgery.

This study was designed to comparatively investigate the relationship between postoperative anterior chamber depth (ACD) and postoperative refraction as well as the differences in the frequency of reverse pupillary block in the context of ISF.

## 2. Materials and Methods

This study was approved by the Institutional Review Board of Tsukazaki Hospital (approval number: 221027) and was conducted in accordance with the 1964 Declaration of Helsinki and its subsequent amendments.

All patients were operated upon by the same highly experienced surgeon at the Tsukazaki Hospital (Himeji, Japan). The subjects were patients who underwent surgery between February 2017 and January 2020. The series included 45 consecutive cases of ISF at 1.5 mm from the corneal limbus (ISF 1.5) when performed between February 2017 and June 2018, in addition to 55 consecutive cases of ISF at 2.0 mm (ISF 2.0) when performed between July 2018 and February 2020. All patients underwent ISF with a 3-port 27-gauge vitrectomy. In the ISF 1.5 group, a total of 12 eyes possessed dislocated crystalline lens and dropped cataract fragments in the vitreous cavity; in addition, 33 eyes possessed a IOL dislocation in the vitreous cavity. In the ISF 2.0 group, there were 14 crystalline lens dislocations and 41 IOL dislocations. All dropped IOLs were divided into three pieces intraocularly and removed; further, no incisional enlargement was performed. The control group included the 50 most recent consecutive cases of cataract operation (phacoemulsification PEA + IOL) and in-the-bag ZCB00V (ZCB), which were both performed around the same time period. All patients underwent surgery in one eye only.

All ISF cases were also performed with a double-needle technique that was administered via a 30 G needle. A temporal sclero-corneal incision of 2.2 mm (right eye: 0°, left eye: 180°) was performed, and the IOL was inserted from this incision. Haptics were fixated within the sclera with the double-needle technique via the use of a flange. All patients underwent an IOL haptic fixation of 90° and 270°. With respect to the ISF 1.5 cases, the haptics were fixated in the sclera 1.5 mm from the corneal limbus. Regarding the ISF 2.0 cases, the haptics were fixated at 2.0 mm from the corneal limbus. In reference to all the ISF procedures, through the 30-gauge needle, a haptic is externalized, then trimmed to the optimal length, and then cauterized before being pushed back into the sclera. The IOL used was NX60 (Santen, Osaka, Japan) in all cases. Prior to surgery, the corneal curvature radius (flat keratometry, Kf, and steep keratometry, Ks) and the axial length were measured via the use of a ZEISS IOLMaster^®^ 700 (Carl Zeiss, Oberkochen, Germany). Swept-source optical coherence tomography was used to measure the ACD (mm) from the corneal endothelium to the front of the IOL with CASIA (Tomey Corporation, Nagoya, Japan). The corrected distance visual acuity and manifest refraction spherical equivalent (MRSE) were measured at 3 months postoperatively (post-op MRSE). Visual acuity was tested through using a Landolt ring chart at a distance of 5 m. Preoperative biometry was used to calculate the predicted refractive values (predicted MRSE) and ACD with the SRK/T formula (predicted ACD) [9]. At 3 months postoperatively, we calculated the difference between the ACD according to the anterior eye optical coherence tomography (post-op ACD) and the predicted ACD (post-op ACD—predicted ACD) as well as through the difference between MRSE and the predicted refractive values according to the SRK/T formula (post-op MRSE—predicted MRSE). We also investigated whether iris capture occurred postoperatively.

The data are expressed as mean ± standard deviation (SD; range). For the purposes of statistical analysis, the chi-square test was used to determine the ISF 1.5 and ISF 2.0 iris capture as well as the *p*-values of <0.05, which were obtained by the Tukey–Kramer test and were used in order to denote statistical significance for all other analyses. In addition, JMP 14.3 was used to perform statistical analyses. The sample size of 55 with respect to ISF 2.0 (post-op MRSE—predicted MRSE (D)) was estimated to detect a 0.11 D difference between the groups, with a significance level of 5% and a power of 80% according to an SD of 0.14 for an average of ISF 2.0 (post-op MRSE—predicted MRSE).

## 3. Results

**Figure 1:**
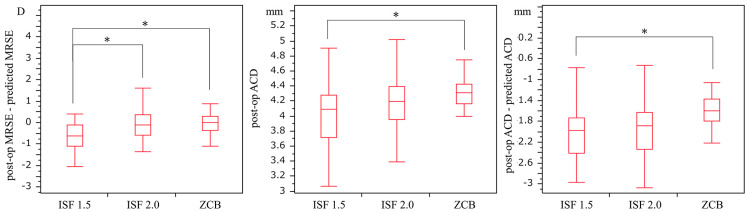
Refraction error and anterior chamber depth (post-op ACD) by fixed position. Significant differences in the post-op MRSE—predicted MRSE were found between the ISF 1.5 and ISF 2.0 groups as well as between the ISF 2.0 and ZCB groups (*p* = 0.01 and *p* = 0.01). There were also significant differences in the post-op ACD between the ISF 1.5 and the ZCB groups (*p* < 0.01). Further, there were significant differences in the post-op ACD—predicted ACD between the ISF 1.5 and ZCB groups as well as between the ISF 2.0 and ZCB groups (*p* < 0.01 and *p* < 0.01). ACD: anterior chamber depth, ISF: intrascleral fixation, and MRSE: manifest refraction spherical equivalent. * Tukey-Kramer test, *p* < 0.05.

Surgery was performed uneventfully in all cases, with no complications being observed. No incidence during surgery of iridocele or iris capture due to intraoperative floppy iris syndrome (IFIS) occurred in any of the cases. No cases of weakness of Zinn’s zonule, such as pseudoexfoliation syndrome, were observed in the patients within the in-the-bag ZCB group. Indeed, none of the patients underwent another surgical procedure postoperatively. Moreover, no sex-related differences were observed among the three groups (*p* = 0.21). The investigation of the patients’ backgrounds revealed the mean ages of 73.13 ± 1.75, 70.67 ± 1.58, and 66.96 ± 1.66 years for the ISF 1.5, ISF 2.0, and ZCB groups, respectively. Thus, a significant difference was observed between the ISF 1.5 and ZCB groups (*p* = 0.03). Furthermore, there were no significant differences among any of the groups for the other patient background parameters, such as axial length, Kf, or Ks (all *p* > 0.05). The post-op MRSEs were −0.76 ± 1.37, −0.57 ± 0.02, and −0.05 ± 0.43 D in the ISF 1.5, ISF 2.0, and ZCB groups, respectively. These results, therefore, indicate a significant difference between the ISF 1.5 and ZCB groups (*p* = 0.0049; Table 1). Indeed, the post-op MRSE—predicted MRSE values were −0.59 ± 0.15, 0.019 ± 0.14, and 0.00 ± 0.05 D in the ISF 1.5, ISF 2.0, and ZCB groups, respectively. As such, this indicated significant differences between the ISF 1.5 and ISF 2.0 groups (*p* = 0.01) as well as between the ISF 1.5 and ZCB groups (*p* = 0.01). At 3 months postoperatively, the post-op ACD was 4.00 ± 0.44, 4.17 ± 0.40, and 4.29 ± 0.25 mm in the ISF 1.5, ISF 2.0, and ZCB groups, respectively, thus suggesting a significant difference between the ISF 1.5 and ZCB groups (*p* < 0.01). The post-op ACD—predicted ACD values were −2.03 ± 0.60, −1.98 ± 0.63, and −1.60 ± 0.33 mm in the ISF 1.5, ISF 2.0, and ZCB groups, respectively, thus implying a significant difference between the ISF 1.5 and ZCB groups (*p* < 0.01). There was also found to be a significant difference between the ISF 2.0 and ZCB groups (*p* ≤ 0.01; Figure 1, Supplemental material S1).

**Figure 2:**
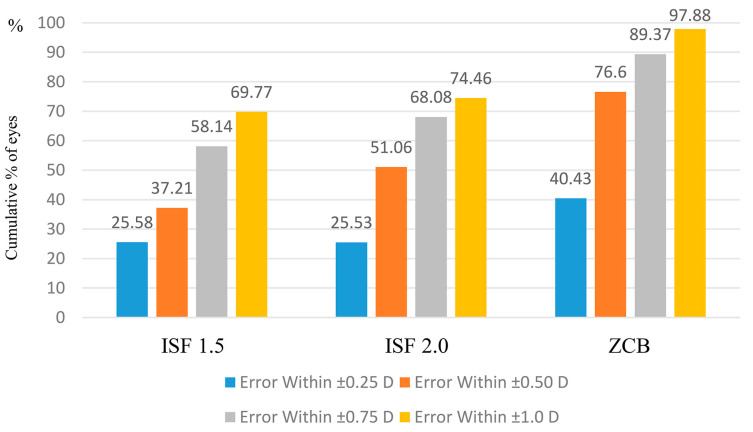
Cumulative refraction error by fixed position. Regarding the cumulative postoperative refractive error within ±1.0 D, according to the post-op MRSE—predicted MRSE, a high result of 97.88% was calculated for the ZCB group, whereas the result was 69.77% for the ISF 1.5 group and 74.46% for the ISF 2.0 group. ISF: intrascleral fixation and MRSE: manifest refraction spherical equivalent.

Regarding the cumulative postoperative refractive error within ± 1.0 D—according to the post-op MRSE—predicted MRSE—the value was highest for the ZCB group at 97.88% (in which the IOL was implanted in the capsule), with 69.77% for the ISF 1.5 group, and 74.46% for the ISF 2.0 group. There was a large difference in the ±0.5 D refractive error, with 89.37% for the ZCB group, 58.14% for the ISF 1.5 group, and 68.08% for the ISF 2.0 group. The cumulative refractive error value of the ZCB group was higher than the other two groups for ±0.25 D, ±0.5 D, and ±0.75 D, and for those within ±1.0 D. Conversely, the value for cumulative postoperative refractive errors over 1.0 D of the two ISF groups were higher than that for the ZCB group (ZCB group: 2.12%, ISF 1.5 group: 30.23%, and ISF 2.0 group: 25.54%; Figure 2).

Iris capture occurred in 7 of the 100 eyes in the ISF group (incidence rate: 7%). It occurred in four eyes (all male) in the ISF 1.5 group (incidence rate: 8.89%) and in three eyes (male: 2 eyes, female: 1 eye) in the ISF 2.0 group (incidence rate: 5.45%). The iris capture did not occur in any of the eyes in the ZCB group (incidence: 0%; Table 2). No significant differences were observed between the ISF 1.5 and ISF 2.0 groups with regard to the occurrence of the iris capture (*p* = 0.52). No correlations were observed for iris capture onset or patient sex between the ISF 1.5 and ISF 2.0 groups (*p* = 0.07 and *p* = 0.87, respectively). Of the patients who developed iris capture, two possessed atopy and one possessed asthma. No findings suggestive of IFIS were obtained intraoperatively; in addition, none of the patients received alpha-1 blockers (Table 2).

## 4. Discussion

We investigated the refractive errors for the IOL fixation position in regard to the ISF and the in-the-bag fixation. The SF 1.5 group possessed a larger myopic surprise, while the ISF 2.0 group possessed less, thus revealing a myopia of 0.6 D in the ISF 1.5 group. The post-op MRSE—predicted MRSE value of the ISF 2.0 group was 0.019 ± 0.14, which was close to the value of 0.00 ± 0.05 D in the ZCB group. The ISF haptic fixation 0.5 mm distal from the corneal limbus may cause a hyperopia of approximately 0.6 D. Moreover, a difference of 0.17 mm was observed between the ACD of the ISF 2.0 group and the ISF 1.5 group. It means that, when fixated, 0.5 mm is further from the corneal limbus, thus entailing the fact that the IOL position became 0.17 mm deeper. A distance of 0.5 mm at the scleral surface away from the limbus results in a 0.17 mm deepening of the IOL position in the vertical direction toward the macular. It is considered that this is due to the fact that the shape of the limbus scleral surface is not perpendicular to the macular but oblique. The mean ACD difference was found to be 0.29 mm between the in-the-bag ZCB and the ISF 1.5 groups as well as 0.12 mm between the ZCB and ISF 2.0 groups. However, little difference in the post-op MRSE—predicted MRSE value was found between the ZCB and ISF 2.0 groups. As NX60 were spherical IOLs and ZCB possessed a −0.27 aspherical aberration, there was only a slight difference found in the post-op MRSE—predicted MRSE value between them [10].

The cumulative refractive error was smaller in the ZCB group than in the two ISF groups. We confirmed that the postoperative refraction prediction is not as precise for ISF as for in-the-bag fixation. A refractive error of ≥1 D was found in approximately 2% of the cases in the ZCB group but in approximately 30% of the cases with regard to the ISF groups. Thus, a significant postoperative refractive error should be expected after ISF. From this research, the following ISF-dedicated calculation formula is established: 

Regarding ISF 1.5 mm

Predicted refraction power (D) = SRK/T expected refraction power − 0.59

Regarding ISF 2.0 mm

Predicted refractive power (D) = SRK/T expected refractive power + 0.019

Although we expected that the postoperative iris capture would occur less frequently in the ISF 2.0 group as the fixation starting point was on the fundus side in this group, no significant difference in the number of cases of iris capture was found between the ISF 1.5 and ISF 2.0 groups. Due to the fact that there was the background to consider, such as lens dislocation, as well as the after high intraocular pressure in the ISF 1.5 and ISF 2.0 groups, significant differences may be observed between the ISF group and ZCB group.

Although there are certain reports on refractive error with respect to the IOL ISF, detailed studies on haptic fixation positions and refraction errors have not been conducted [7,8]. It is reported that there is a refraction error when compared to the SRK/T formula with an average of −0.38D, but the relationship with the fixed position of the IOL is unknown. The fixed position of the haptics differs depending on the operator and the surgical environment. In other words, if the fixed position of the haptics is different, the refractive error and other factors should be different. However, there is no report that has examined this in detail. In addition, the occurrence of iris capture is a problem in ISF, but the incidence of this is unknown. For the first time in this paper, we reported the refractive error and ACD at fixed IOL positions of 1.5 mm and 2.0 mm. In this study, it was also shown that pupil capture cannot be prevented, even if the fixed position is as deep as a 2.0 mm ISF. This study also revealed that the difference in refraction and the ACD is between 1.5 mm and 2.0 mm in the haptic fixed positions.

Overall, many of the myopic eyes possessed low IOL power. ACD, which indicated the IOL fixation position, was not abnormally deep or shallow for any cases in which iris capture occurred. Although six of the seven patients with iris capture were males, our analysis did not reveal a sex-related difference. Moreover, although the patients’ medical history did not reveal that any of the patients had been administered alpha-1 blockers, which can cause IFIS, our results may have been affected by the presence of patients with low iris tension or similar conditions. Indeed, the tilt of the IOL, the difference in iris rigidity, and the decentration of the IOL may have affected iris capture.

No cases of intraoperative wound capture of the iris were observed. Furthermore, none of the IFIS findings were observed intraoperatively, but there may be a certain degree of difference in iris stiffness depending on the case. The reason iris trapping occurs is considered to be related to the difference in iris rigidity, the slight inclination of the IOL, and the depth of the IOL. Although the severity of the IFIS syndrome was found to be low, cases with low iris tension or similar conditions may have affected our results. Indeed, the iris capture cannot be prevented by changing the ISF position from 1.5 mm to 2.0 mm.

It must be noted that this study possesses certain limitations. As this was a retrospective study, our results may have been affected by group classifications, patient backgrounds, or limited sample size. A significant difference in age between ISF1.5 and ZCB as well as a significant difference in CDVA between ISF1.5, ISF2.0, and ZCB may affect this study. Preoperative glaucoma, intraocular pressure, dry eye, pupil diameter, corneal condition, etc., are all also thought to affect postoperative visual acuity and the accuracy of predicted refraction values. As the number of cases increases, it is conceivable that the refractive error and ACD will approach highly reliable values.

## 5. Conclusions

In conclusion, ISF 2.0 possessed 0.6D hyperopia and a 0.17 mm deeper lens position than what was obtained with ISF 1.5. The refractive error of ISF 2.0 was less than that of ISF 1.5. Indeed, no significant iris capture onset was noted between ISF 2.0 and ISF 1.5. Furthermore, when performing ISF, it is suggested that the postoperative refractive error may be improved by utilizing the calculation from the refractive error that was obtained in this study, depending on the position of where the IOL is fixed.

## Figures and Tables

**Table 1:** Clinical features of the study eyes.

	ISF 1.5		ISF 2.0		ZCB		*p*		
	Mean	SD	Mean	SD	Mean	SD	ISF 1.5 vs. ISF 2.0	ISF 1.5 vs. ZCB	ISF 2.0 vs. ZCB
n	45		55		50				
Female	19		16		20				
Age (years)	73.13	1.75	70.67	1.58	66.96	1.66	0.55	0.03 *	0.24
Kf (mm)	7.69	0.27	7.75	0.34	7.82	0.28	0.11	0.59	0.51
Ks (mm)	7.55	0.04	7.57	0.04	7.67	0.04	0.96	0.12	0.16
Axial length (mm)	23.98	0.25	24.38	0.22	24.15	0.23	0.46	0.88	0.75
CDVA (LogMAR(Snellen))	0.3(20/39.9)	0.53	0.24(20/34.8)	0.55	−0.12(20/15.2)	0.1	0.79	<0.01 *	<0.01 *
post-op MRSE (D)	−0.76	1.37	−0.57	1.18	−0.05	0.43	0.65	<0.01 *	0.05

Upon comparing the three groups, a significant difference was observed between the ISF 1.5 mm group and the ZCB00V group in terms of age (*p* = 0.03). No significant difference was observed in the other categories of preoperative data, nor in the K1, K2, and axial axes. The postoperative-corrected visual acuity CDVA showed a significant difference between the ISF 1.5 mm group and the ZCB00V group (*p* < 0.01). Regarding post-op MRSE, a significant difference was observed between the ISF 1.5 mm group and the ZCB00V group (*p* < 0.01). * Tukey–Kramer test (*p* < 0.05). CDVA: corrected distance visual acuity, ISF: intrascleral fixation, MRSE: manifest refraction spherical equivalent, K1: flat keratometry, and K2: steep keratometry.

**Table 2:** Background of the iris capture patients.

Sex	Age	L or R	ISF	IOL Power	Axial Length	Kf	Ks	Post-Op ACD	Medical History
M	55	R	ISF 1.5	10	27.76	7.95	7.84	3.92	Asthma
M	75	R	ISF 1.5	10	26.45	7.43	7.26	3.99	
M	66	L	ISF 1.5	21	23.19	7.55	7.37	4.24	
M	37	L	ISF 1.5	16.5	28.32	8.31	9.07	3.51	Atopic dermatitis
M	52	R	ISF 2.0	10	25.59	7.13	6.92	4.52	
F	63	L	ISF 2.0	10	30.44	8.33	8.09	4.16	
M	47	L	ISF 2.0	21.5	23.96	7.89	7.89	4.07	Atopic dermatitis, diabetic mellitus

The iris capture occurrences were not significantly different at the ISF fixed position. The postoperative ACD (post-op ACD) did not differ significantly regarding the iris capture occurrence. One case possessed asthma and two cases possessed atopic allergies. ACD: anterior chamber depth, F: female, IOL: intraocular lens, ISF: intrascleral fixation, M: male, Kf: flat keratometry, and Ks: steep keratometry.

## Data Availability

The datasets generated and/or analyzed during the current study are available from the corresponding authors on reasonable request.

## Supplementary Materials

The following supporting information can be downloaded at: https://www.mdpi.com/article/10.3390/jcm12051815/s1.
